# Mode of action of abatacept in rheumatoid arthritis patients having failed tumour necrosis factor blockade: a histological, gene expression and dynamic magnetic resonance imaging pilot study

**DOI:** 10.1136/ard.2008.091876

**Published:** 2008-09-04

**Authors:** M H Buch, D L Boyle, S Rosengren, B Saleem, R J Reece, L A Rhodes, A Radjenovic, A English, H Tang, G Vratsanos, P O’Connor, G S Firestein, P Emery

**Affiliations:** 1 Academic Unit of Musculoskeletal Disease, University of Leeds, Leeds, UK; 2 Center for Innovative Therapy, Biomarker Laboratory, University of California, San Diego, California, USA; 3 Academic Unit of Medical Physics, University of Leeds, Leeds, UK; 4 Bristol-Myers Squibb, Princeton, New Jersey, USA; 5 Department of Radiology, Leeds General Infirmary, Leeds, UK

## Abstract

**Objectives:**

Abatacept is the only agent currently approved to treat rheumatoid arthritis (RA) that targets the co-stimulatory signal required for full T-cell activation. No studies have been conducted on its effect on the synovium, the primary site of pathology. The aim of this study was to determine the synovial effect of abatacept in patients with RA and an inadequate response to tumour necrosis factor alpha (TNFα) blocking therapy.

**Methods:**

This first mechanistic study incorporated both dynamic contrast-enhanced (DCE) magnetic resonance imaging (MRI) and arthroscopy-acquired synovial biopsies before and 16 weeks after therapy, providing tissue for immunohistochemistry and quantitative real-time PCR analyses.

**Results:**

Sixteen patients (13 women) were studied; all had previously failed TNFα-blocking therapy. Fifteen patients completed the study. Synovial biopsies showed a small reduction in cellular content, which was significant only for B cells. The quantitative PCR showed a reduction in expression for most inflammatory genes (Wald statistic of p<0.01 indicating a significant treatment effect), with particular reduction in IFNγ of −52% (95% CI −73 to −15, p<0.05); this correlated well with MRI improvements. In addition, favourable changes in the osteoprotegerin and receptor activator of nuclear factor kappa B levels were noted. DCE–MRI showed a reduction of 15–40% in MRI parameters.

**Conclusion:**

These results indicate that abatacept reduces the inflammatory status of the synovium without disrupting cellular homeostasis. The reductions in gene expression influence bone positively and suggest a basis for the recently demonstrated radiological improvements that have been seen with abatacept treatment in patients with RA.

Understanding of disease pathogenesis in rheumatoid arthritis (RA) has led to novel approaches in targeted drug development. Despite the demonstrated success of tumour necrosis factor (TNF) antagonists, up to 50% of patients have an inadequate response to TNF blockade therapy.^1^^–^4 This observation has fuelled the search for alternative targeted approaches.

Abatacept is a recombinant fusion protein comprising the extracellular domain of human cytotoxic T-lymphocyte antigen 4 and a fragment of the Fc domain of human IgG1. It acts by competing with CD28 for binding to CD80/CD86, modulating the second co-stimulatory signal required for full T-cell activation.^5^ ^6^

Abatacept has demonstrated benefits in patients with RA and an inadequate response to methotrexate^7^ that are comparable to those observed in studies of TNF blockade, with efficacy also confirmed in the particularly resistant group of patients who have failed TNF blockade therapy.^8^

There is limited information on the impact of co-stimulation modulation on the synovium. The objective of this first mechanistic study was to determine the synovial effect of abatacept in a TNF antagonist-resistant group of patients. A novel and validated method of gene expression analysis was employed in combination with immunohistochemistry to evaluate the changes in synovial pro-inflammatory cytokine gene expression and cell populations, respectively, with evaluation of magnetic resonance imaging (MRI) changes before and after abatacept therapy.

## PATIENTS AND METHODS

This was a collaborative, prospective, open-label study between the Academic Unit of Musculoskeletal Disease, University of Leeds and the Center for Innovative Therapy, University of California San Diego, sponsored by Bristol-Myers Squibb. Leeds research ethics committee approval was obtained before study initiation. The study was conducted in accordance with the ethical principles of the Declaration of Helsinki. All patients provided written informed consent. The US Food and Drug Administration registration number for this clinical trial is NCT00162201.

### Patients

All patients were recruited from the Leeds Biologic Clinic, had a diagnosis of RA, as defined by the 1987 American College of Rheumatology criteria^9^ and had currently or previously failed a TNF-blocking therapy. TNF blockade inefficacy was defined as failure of the disease activity score 28 (DAS28) to improve by 1.2 or more after 3 months of therapy as per British Society of Rheumatology guidelines.^10^ Patients were also required to have evidence of active disease defined by a DAS28 of more than 5.1 and a tender and swollen knee joint identified as a target joint for arthroscopy.

Exclusion criteria included: patients with evidence of active tuberculosis; previous tuberculosis; chest *x* ray granuloma or tuberculosis exposure with a mantoux reading of 5 mm or greater if no previous history of bacillus Calmette–Guérin (BCG), or 10 mm or more if patients had previously received BCG. Pregnant or lactating women; patients with a history of septic arthritis in the last year and those with severe co-morbidity, including a history of recurrent infections, were also excluded.

### Concomitant therapy

Background disease-modifying antirheumatic drugs for at least 3 months and at stable doses for at least 28 days before the first dose of abatacept were required (methotrexate (subcutaneous/intramuscular), hydroxychloroquine and sulphasalasine permitted). Low-dose stable corticosteroids and/or stable non-steroidal anti-inflammatory drugs were allowed. Patients who were currently receiving TNF-blocking agents were required to have discontinued etanercept and adalimumab for at least 28 days or infliximab for at least 60 days before day 1.

### Study schedule

Following successful screening at a maximum of day −28, patients underwent clinical evaluation to confirm the DAS28 (erythrocyte sedimentation rate; ESR) score and also dynamic contrast-enhanced (DCE) MRI and arthroscopy of the target knee joint between days −6 and 0. Arthroscopy was performed within 2 days of the DCE–MRI scan. All patients received abatacept by intravenous infusion according to baseline weight (<60 kg received 500 mg, 60–100 kg inclusive received 750 mg and >100 kg received 1000 mg). Six infusions were administered, each over approximately 30 minutes on days 1, 15, 29, 57, 85 and 113.

Clinical assessments were repeated at day 57. Following completion of treatment, patients had further clinical evaluation for DAS28 (ESR) calculation, DCE–MRI and arthroscopy of the target knee joint between days 120 and 127. Below is a summary of methods (complete details included in the supplementary information published online only).

### Arthroscopy and synovial biopsy

Patients underwent medical arthroscopy of a swollen knee joint before commencing abatacept therapy and after completion of treatment as described above with multiple synovial biopsies obtained. The arthroscopist (RJR) was blinded to the clinical response.

### Immunohistochemistry

The cell types analysed by immunohistochemistry included: lining layer (LL) and sub-lining layer (SL) CD3+, CD154 (CD40L)+ and CD4+ T cells; CD80+ and CD86+ antigen presenting cells; CD20+ and CD79+ B cells; CD55+ synovial fibroblasts; CD54+ cells (intercellular adhesion molecules); CD68+ synovial macrophages; and CD11b+ neutrophils, macrophages and dendritic cells. A standard staining procedure using ChemMate (DakoCytomation, Glostrup, Denmark) was used. 3,3′-Diaminobenzidine was used to develop colour.

### Microscopic analysis

Sections were randomly analysed and the histological features scored in blinded fashion (by MHB and AE) using a validated semiquantitative scoring system.^11^

### Quantitative real-time PCR

Methods for messenger RNA analysis have previously been described.^12^ Pooled biopsy fragments were used to synthesise complementary DNA. The TaqMan PCR method was undertaken for gene expression analysis of IL-1, IL-6, matrix metalloproteinase (MMP) 1, MMP-3 and IFNγ. Results were expressed in relative expression units.^12^

### DCE–MRI and image processing

Dynamic (during and after contrast agent gadolinium diethylenetriaminepentaacetic acid) MRI of the knee was performed using a Philips 1.5T Gyroscan ACS-NT whole-body scanner (Philips Medical Systems, Best, The Netherlands) with a Philips quadrature knee coil.

### Image analysis

Commercial software (Analyze, Mayo Clinic, New York, USA) and software developed in-house^13^ were used to calculate values for the maximal enhancement (ME) and the initial rate of contrast enhancement (IRE) on a pixel-by-pixel basis. ME and IRE provide an assessment of the synovial microcirculation.

### Measurements of MRI parameters

#### Global

ME and IRE were measured in regions of interest (ROI) defining the extent of the synovitis using the image analysis software at a level of 10 pixels below the tibial plateau to the top of the suprapatellar pouch (SPP) (regions of SPP, cartilage–pannus junction and a distant site in the SPP).^14^ Data analysis was performed by a blinded investigator (LAR).

### Statistical analysis

#### Immunohistochemistry

Median baseline semiquantitative scores for cell populations were calculated for responders, non-responders and the overall patient population. The Wilcoxon signed rank test was applied to determine significant changes in the parameters following abatacept therapy. As this was a pilot study comprising relatively small numbers, Bonferroni correction was not applied. Any significant changes observed posttreatment would represent trends for potential further evaluation.

#### Gene expression data

Summary statistics and their mean changes and mean percentage changes from baseline were provided for mRNA levels of pro-inflammatory markers for responders, non-responders and the overall patient population. Mean and mean percentage changes of log-transformed PCR data were expressed as geometric mean and the geometric mean percentage changes, respectively. Point estimates and the two-sided 95% CI on the log scale were exponentiated to obtain estimates on the original scale.

The treatment effect on all pro-inflammatory gene expression was tested simultaneously using mean treatment effects and their covariance matrix in post-hoc analyses based on Wald statistics; a p value of less than 0.01 indicates a significant treatment effect on the parameters simultaneously.

#### Magnetic resonance imaging

Median values of ME and IRE were calculated by dividing the sum IRE or ME results for each ROI by the number of pixels enhancing within the ROI. Median values of percentage change from baseline for ME and IRE were calculated. The post-hoc Pearson correlation test evaluated for a relationship between the gene expression data and MRI outcomes.

## RESULTS

### Baseline patient demographics and disease characteristics

Sixteen patients were recruited (13 female; mean age 53.8 years); 15 completed the study (one patient dropped out due to elective toe surgery) (table 1). Twelve of the 15 patients had received more than one TNF-blocking agent, 12 were on concomitant methotrexate, 75% of patients were rheumatoid factor positive. Mean baseline DAS28 was 7.1 (SEM 0.22), with mean swollen and tender joint counts of 14.0 (SD 6.0) and 19.2 (SD 7.2), respectively. High baseline inflammatory markers were noted with a mean C-reactive protein (CRP) level of 5.8 mg/dl (SD 5.6) and ESR of 58.9 mm/h (SD 27.0).

**Table 1:** Baseline patient demographics and disease characteristics

	All patients (n = 16)
Patient demographics	
Age, years	53.8 (11.5)
Gender, % female	81.0
Race, % white	94.0
Weight, kg	72.6 (17.3)
Disease characteristics	
Rheumatoid factor positive* (%)	75.0
CRP, mg/l	5.8 (5.6)
ESR, mm/h	58.9 (27.0)
VAS, 100 mm	66.5 (15.5)
Baseline DAS28	7.1 (0.9)
Baseline tender joint count, /28	14.0 (6.0)
Baseline swollen joint count, /28	19.2 (7.2)

Data are presented as means (SD) unless otherwise stated; *n  =  15; CRP, C-reactive protein; DAS28, disease activity score 28; ESR, erythrocyte sedimentation rate; VAS, visual analogue scale for patient global assessment of disease activity.

### Clinical response

A gradual (significant) decline in overall DAS28 was recorded, with mean DAS28 at baseline, day 57 and day 120 of 7.1 (SEM 0.22; n  =  16), 6.13 (SEM 0.34; n  =  14) and 5.77 (SEM 0.37; n  =  15), respectively (p<0.01). Nine of the 15 patients (60%) completing the study demonstrated a clinical response to abatacept treatment (reduction in DAS28 of ⩾1.2). Ten of the 15 patients demonstrated a “moderate” EULAR response; one patient had a “good” EULAR response. A median (25th, 75th percentile) percentage reduction in CRP of 66.7% (90.0%, 44.4%) was noted from baseline. No significant differences in the baseline clinical features were observed between the clinical response and non-response groups (data not shown).

### Immunohistochemistry

**Figure 1:**
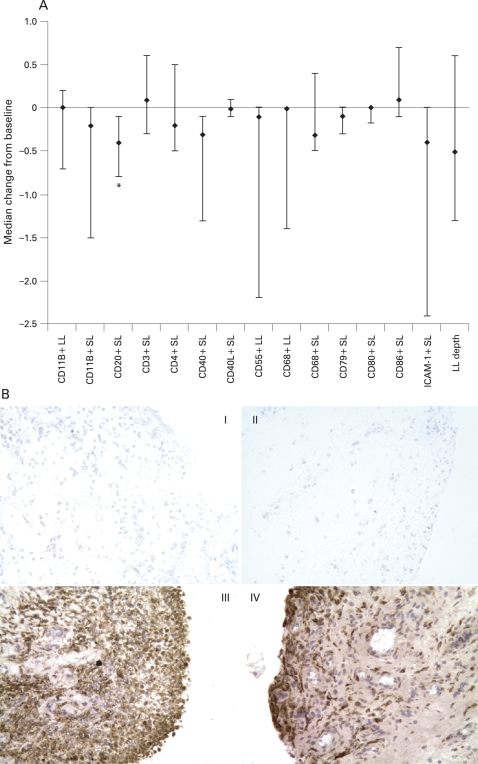
(A) Median change in synovial expression of cellular infiltrate after 4 months of treatment with abatacept. Multiple synovial biopsies were obtained from representative inflamed sites and stained with antibodies to a range of synovial markers. Synovial lining layer (LL) and sub-lining layer (SL) expression of each marker was scored semi-quantitatively on a five-point scale (0, minimal infiltration; 4, maximal infilatration) and a median percentage change from baseline calculated. *p<0.05. Error bars represent 25th and 75th percentiles; the number of patients analysed was 11 in all cases. (B) Example of CD68 expression in paired synovial tissue samples from a patient with rheumatoid arthritis treated with abatacept. Panels I and II are the negative controls for the patient at baseline and post-abatacept, respectively. Panels III and IV demonstrate macrophage (CD68) staining at baseline and post-abatacept, respectively. Considerable LL and SL CD68 expression is observed in the baseline biopsy; expression continues to be marked post-abatacept. ICAM-1, intercellular adhesion molecule type 1.

Small reductions in some of the synovial markers analysed were observed following abatacept treatment (fig 1A). A modest but significant (p<0.05) reduction in SL CD20 was noted from baseline (0.83) to day 120 (0.42), with a median change (25th, 75th percentile) of −0.4 (−0.8, −0.1). A trend for reduction in the SL baseline and posttreatment values was observed for CD11b (2.35 and 1.93), CD68 (2.52 and 2.33), CD40 (1.04 and 0.48), CD79 (0.21 and 0.08) and intercellular adhesion molecule type 1 (3.16 and 2.18). Trends in LL reductions from baseline to posttreatment, respectively, were also seen for CD68 (3.58 and 3.0) and CD55 (2.93 and 2.28) with a small reduction in LL depth from 1.45 to 0.96 (fig 1A), although these changes were highly variable. Figure 1B shows CD68 expression in representative paired synovial tissue samples, in which the degree of expression is largely unchanged following abatacept treatment.

### Gene expression analysis

**Figure 2:**
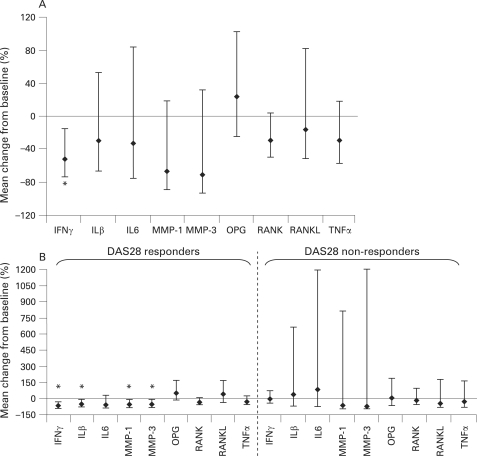
Geometric mean percentage change in synovial gene expression in patients after 4 months of treatment with abatacept in (A) all patients combined (n  =  14^†^), (B) disease activity score 28 (DAS28) responders (n  =  8^†^) versus DAS28 non-responders (n  =  6^†^). RNA was isolated from the synovium using chloroform extraction and reverse transcribed to cDNA. mRNA levels of tumour necrosis factor alpha (TNFα), IL-1β, IL-6, matrix metalloprotease (MMP) 1, MMP-3, IFNγ, receptor activator of nuclear factor kappa B (RANK), RANK ligand (RANKL) and osteoprotegerin (OPG) were quantified using TaqMan quantitative PCR analysis. Data are expressed as the geometric mean percentage change in gene expression relative to baseline. Error bars represent 95% CI. *95% CI do not include zero. ^†^Two patients (one responder and one non-responder) had IFNγ levels below the detectable limit at baseline and one patient (non-responder) had IL-6 levels below the detectable limit at baseline; these patients were excluded from analysis for these cytokines. Wald statistics indicate a significant treatment effect of abatacept on IFNγ, IL-1β, IL-6, MMP-1 and MMP-3 simultaneously (p<0.01).

In the total population, a significant (p<0.05) geometric mean percentage change in IFNγ gene expression of −52% (95% CI −73 to −15) was observed (fig 2A). In addition, reductions (non-significant) in IL-1β, IL-6, MMP-1, MMP-3 and TNFα gene expression were noted. Interestingly, a decreasing trend in receptor activator of nuclear factor kappa B (RANK) and RANK ligand (RANKL) gene expression was seen in association with an increase in osteoprotegerin gene expression (fig 2A).

The mean treatment effects of IFNγ, IL-1β, IL-6, MMP-1 and MMP-3 (−0.32, −0.27, −0.23, −0.62 and −0.67, respectively) and their covariance matrix were estimated. Using Wald statistics, a value of p<0.01 was noted, indicating a significant treatment effect of abatacept on these parameters simultaneously.

Analysis of the responder (reduction in DAS28 score of ⩾1.2; n  =  7) and non-responder groups demonstrated significant reductions (p<0.05) in IFNγ gene expression in the responder group only, with a mean percentage change of −69% (95% CI −87 to −27) (fig 2B). Significant reductions (p<0.05) in additional parameters were also observed, namely mean percentage change in gene expression of IL-1β (−53%; 95% CI −77 to −6), MMP-1 (−61%; 95% CI −83 to −11) and MMP-3 (−59%; 95% CI −82 to −5) (fig 2B). In addition, osteoprotegerin expression increased posttreatment (geometric percentage mean change of 53% in responders).

Immunohistochemistry demonstration of reduced synovial B-cell expression was confirmed by a reduction in CD19 transcript levels (data not shown) although IgM transcript levels were unchanged.

### Magnetic resonance imaging

**Figure 3:**
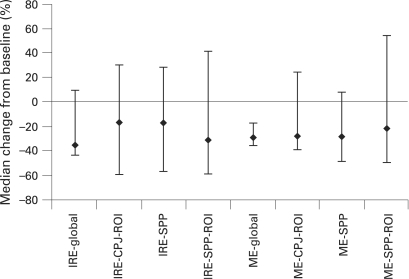
Median percentage change in key magnetic resonance imaging (MRI) measures. MRI of the knee was performed, and measures of the initial rate of enhancement (IRE) and maximal enhancement (ME) made in the global region as well as in regions of interest (ROI) at the cartilage–pannus junction (CPJ) and the suprapatellar pouch (SSP). Values for the ME and IRE are calculated from the sum of the enhancing pixels in each region of interest, expressed as arbitrary units. Error bars represent 25th and 75th percentiles.

**Figure 4:**
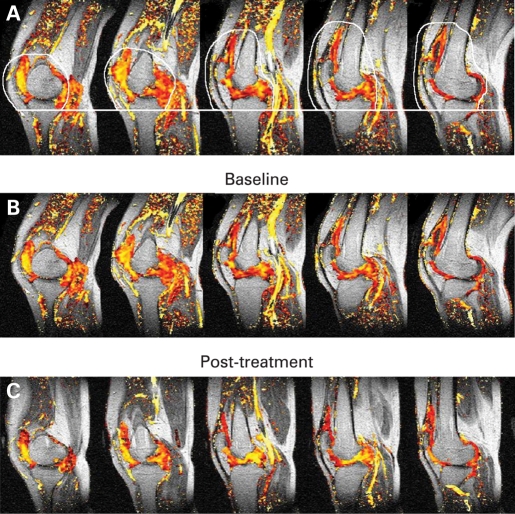
Five characteristic dynamic gadolinium-enhanced magnetic resonance imaging sagittal scans acquired from the knee with superimposed colour data showing values of initial rate of enhancement (IRE) across synovial space following gadolinium enhancement—pixels shown in yellow represent high IRE values whereas red show relatively lower values. Image (A) shows global region of interest outlined in white, which defines the extent of the synovitis at a level of 10 pixels below the tibial plateau to the top of the suprapatellar pouch, avoiding enhancement from muscle and arteries in each of the five sagittal images. Images (B) and (C) are an example of pre and posttreatment, respectively.

**Figure 5:**
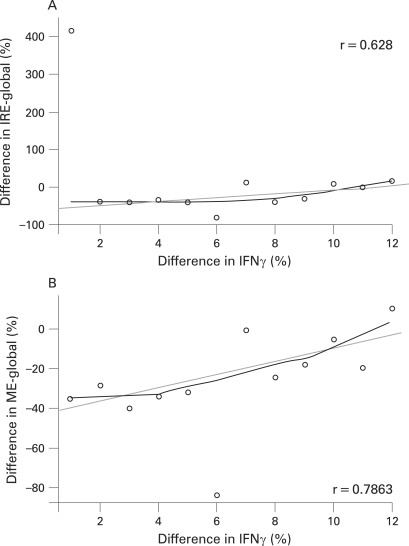
The relationship between the IFNγ gene expression and magnetic resonance imaging (MRI) outcomes. Pearson correlation analyses were carried out to evaluate the relationship between gene expression data and MRI outcomes. A good correlation between IFNγ and the initial rate of enhancement (IRE)-global, as well as maximal enhancement (ME)-global was observed.

For the total population, a significant median percentage reduction from baseline in ME-global of −28.5% (p = 0.001) was observed with a reduction also in the IRE-global of −34.9%. Eleven of 15 patients (73.3%) showed a reduction in IRE-global and 14 of 15 patients (93.3%) in ME-global pre to posttreatment, indicating a reduction in synovitis. There was a greater reduction in IRE-global in clinical responders compared with non-responders (median percentage change −42% and −10%, respectively); however, there was no significant difference in changes in ME-global between clinical responders and non-responders. The data for the smaller ROI were more variable and no consistent changes were observed across the whole study population. Figure 3 presents the median percentage change from baseline in key MRI measures for the total population. Results include DCE–MRI parameters within the global region as well as ROI at the CPJ and the SPP. Figure 4 shows representative example of a DCE–MRI image with the area used to compute global IRE and ME and images showing changes in DCE–MRI pre and post-abatacept treatment. Pearson correlation analyses were undertaken to evaluate for a relationship between the gene expression data and MRI outcomes. A good correlation between IFNγ and IRE-global, as well as ME-global, was observed (fig 5; r  =  0.628 and 0.7863).

## DISCUSSION

Abatacept, a modulator of T-cell co-stimulation, has demonstrated efficacy and an acceptable safety profile in patients with RA and an inadequate response to methotrexate^7^ and in the challenging group of patients resistant to TNF blockade.^8^ Little information is available on the changes that occur with abatacept on the synovium, the primary site of disease. This study is the first to evaluate the mechanistic effects of abatacept, with particular focus on TNF blockade failures. The findings demonstrate contrasting synovial changes: a clear generalised reduction in synovial pro-inflammatory gene expression (particularly IFNγ), but with little change in the composition of the synovial infiltrate. In particular, the reduction in synovial IFNγ is consistent with a T-cell-dependent mechanism of action. The improvement observed in imaging outcomes with reduced tissue perfusion and vascular permeability (indicative of inflammation and correlated with synovial volume)^15^ complements these findings further.

Improved understanding of T-cell biology, with recognition of antigen-independent T-cell influences strongly supports the involvement of T cells in RA pathogenesis.^16^ ^17^ The relative inefficacy of T-cell depletion strategies led to the concept of modulating as opposed to depleting T-cell function, with the targeting of one of the more prominent T-cell co-stimulatory signals, the CD28:CD80/CD86, by interaction with abatacept.

This hypothesis-generating study specifically concentrated on patients failing one or more TNF antagonists. Several studies to date have utilised synovial immunohistochemistry to evaluate the effects of therapeutic intervention.^11^ ^18^^–^20 In this collaborative study, we chose to complement this technique with a validated method of real-time quantitative PCR utilising pooled biopsy fragments for gene expression analysis. DCE–MRI was included to correlate the clinical and synovial data with refined synovial imaging.

Sixty per cent of patients treated with abatacept demonstrated a significant improvement in disease activity (DAS28 reduction of ⩾1.2). Although the DAS28 remained high, suggesting only a modest clinical improvement, these data are consistent with a resistant cohort and mimic the published trial data that formed the basis for the approval of abatacept and rituximab in TNF blockade failures.^8^ ^21^

Several key points can be learned from this study. Only a modest reduction in cellular infiltrate composition was observed with abatacept, and surprisingly this was found mainly in the B-cell population; this is in marked contrast to similar studies conducted to assess the effect of TNF blockade, which revealed significant reductions in cellularity (including macrophage populations).^11^ ^18^^–^20 The modest clinical improvement could account for these findings; however, this difference is not necessarily a surprise when one considers the nature of the targets. TNFα is a pleiotropic cytokine, the effects of which include promoting the recruitment of inflammatory infiltrate and the induction of adhesion molecules. Conversely, abatacept does not directly target the T cell; by targeting the more classic co-stimulatory signal, selective modulation of T-cell activation occurs. Notably, there was no significant decrease in the number of SL macrophages even though others have proposed CD68+ cell depletion as a primary biomarker predictive of clinical response.^22^ We would suggest the importance of considering novel mechanisms of action despite the findings contrasting with previously held assumptions. Semiquantitative as opposed to quantitative analysis could be argued also to account for the relative negative findings, although previous studies have demonstrated the ability of this method to demonstrate significant changes after effective treatment.^11^ ^23^^–^26 In addition, the significant reduction in CD20 in the responder group suggests the sensitivity of the method.

This observed reduction in CD20+ cells also suggests more of a modulating as opposed to cellular-depleting effect of abatacept; In fact, human cytotoxic T-lymphocyte antigen type 4 immunoglobulin binds CD80/CD86 on B cells,^27^ as well as T cells. Further detailed studies are required to explore this observation, including an evaluation of synovial germinal centre formation and other markers of B-cell biology.

In contrast to the histology, the gene expression study demonstrated diminution in pro-inflammatory gene expression, with significant reductions in MMP-1 and MMP-3, and smaller reductions in TNFα, IL-1β and IL-6. This is consistent with a previous study that also illustrated a reduction in MMP gene expression following effective treatment.^28^ MMP expression, being an “integration” of all of the pro-inflammatory signals, would explain the more pronounced reduction in MMP gene expression compared with the individual cytokines. These findings are emphasised with a demonstration of treatment effect on pro-inflammatory gene expression simultaneously (Wald statistic). The significant reduction in IFNγ gene expression, however, certainly suggests an effective functional effect of T-cell modulation on the synovium. Although comprising small numbers, comparison of the abatacept response and non-response groups further verifies this observation, with significant reductions in MMP-1, MMP-3 and IL-1β gene expression, in addition to IFNγ gene expression noted in the response group. A reduction in CD19 transcript levels was consistent with the immunohistochemistry findings; interestingly, IgM transcripts did not change, suggesting the B-cell effect is different to that noted with anti-CD20 both in magnitude and character.^29^

Another interesting finding from this study was the divergent (and advantageous) trend observed between RANK/RANKL and osteoprotegerin gene expression. Osteoprotegerin inhibits the effects of the RANK/RANKL interaction by competing with RANK, thereby functioning as a soluble decoy receptor for RANKL. The reduction in RANK/RANKL expression with concomitant osteoprotegerin upregulation is consistent with a more regulatory influence on osteoclast cell differentiation and bone resorption. Whereas the exact mechanism underlying this observation is unclear, it correlates well with the radiological improvement that has been reported with abatacept therapy.^7^

DCE–MRI was undertaken pre and post-abatacept therapy, with measurement of rates of contrast enhancement (IRE and ME, shown to correlate with inflammatory activity and blood vessel density)^15^ confirming a general reduction in the IRE and ME values of all regions evaluated, with a significant reduction in ME-global. The inability to detect a significant reduction in the global IRE and lack of consistent change in the small ROI is likely to reflect the relatively small sample size, response heterogeneity and/or reproducibility of DCE–MRI measurements. In addition, the differences between responders and non-responders make it harder to detect a reduction in the group as a whole. Nevertheless, the correlation between the MRI parameters and IFNγ gene expression further suggests the synovium as a site of action for abatacept.

Several limitations must also be considered in the interpretation of these data. First, as a result of ethical considerations we did not include a placebo arm. Second, the study population was small and comprised patients with severe disease who were refractive to previous treatment; other similar studies in this population have shown limited changes and a large variation in responses.^29^ ^30^ Finally, it is possible that the primary impact of abatacept may be at another site, such as secondary lymphoid organs.

In summary, we report the first mechanistic study incorporating DCE–MRI and tissue for immunohistochemistry and quantitative real-time PCR analyses before and after abatacept therapy. These results demonstrate a treatment effect on inflammatory mediators simultaneously, with convincing effect on IFNγ, confirming a functional T-cell effect. This contrasts with a modest histological change, suggesting deactivation rather than depopulation of the synovium, although a reduction in synovial “quantity” is observed as evidenced by the MRI. Finally, this reduction in inflammation correlates well with the effect on IFNγ. Overall, the study findings are in keeping with the immunomodulatory nature of selective co-stimulation. The changes in bone biology and B cells add an interesting dimension to the potential effect and mechanism of action of co-stimulation modulation. Further lines of investigation in a larger patient population should further elucidate the synovial effects of co-stimulation modulation with abatacept.
